# Genotypic variability in cotton's transpiration response under progressive soil drying

**DOI:** 10.3389/fpls.2024.1451993

**Published:** 2024-12-04

**Authors:** Katrina J. Broughton, Eleanor L. Bate, Corey W. Cutler, Christopher N. Allen, Alan J. E. Thompson, Warren C. Conaty

**Affiliations:** Commonwealth Scientific and Industrial Research Organisation, Agriculture and Food, Narrabri, NSW, Australia

**Keywords:** abiotic stress, drought, fraction of transpirable soil water, physiology, water deficit

## Abstract

**Introduction:**

Crop yields in food and fibre production systems throughout the world are significantly limited by soil water deficits. Identifying water conservation mechanisms within existing genotypes is pivotal in developing varieties with improved performance in water-limited conditions. The objective of this study was to screen Australian germplasm for variability in the transpiration response to progressive soil drying using a glasshouse dry-down experiment. It tests the hypothesis that water conservation traits may provide tolerance to water stress, particularly when combined with other drought stress traits.

**Methods:**

Three glasshouse experiments were conducted to identify whether there are differences in the fraction of transpirable soil water (FTSW) threshold values for transpiration decline among six cotton genotypes. We also assessed whether genotype dependent responses to progressive soil drying are evident from leaf-level physiology, by measurement of gas exchange parameters.

**Results:**

Significant variation in the FTSW threshold for transpiration decline between six genotypes was found, ranging from 0.13 to 0.29. Genotypic variation in the response to soil drying was also observed from leaf level physiology, with reductions in stomatal conductance and photosynthetic rate coinciding with when the FTSW threshold was reached.

**Discussion:**

Genotypes that limit transpiration at high FTSW can conserve water earlier in the season to maintain productivity during extended dry periods. Therefore, these genotypes may provide physiological traits that improve productivity in water-limited environments. This research is important as rainfall and water resources for irrigated agriculture are predicted to decline. The development of drought tolerant germplasm for the Australian cotton industry will be beneficial in the projected increasingly frequent limited water environments resulting from a changing climate.

## 1 Introduction

Soil water deficits cause significant yield losses in crop production systems. In Australia, water stress is a significant abiotic stress factor limiting cotton yields (Hearn, 1979, Hearn, 1994; Roth et al., 2013). Anthropogenic climate change and a burgeoning global population are further exacerbating these water scarcity issues. Greater variability in precipitation, more frequent droughts and rising global temperatures predicted to occur over the next century will see a reduction in the availability and security of water resources for irrigated agriculture (Williams et al., 2018). In addition to climatic extremes, existing water deficits are being intensified by greater competition for water resources between irrigators and domestic users, and increased environmental allocations (Pimentel et al., 2004). Whilst the Australian cotton industry has traditionally maintained high yields via irrigation water supply, as water resources diminish, expansion into partially irrigated and rainfed systems will be necessary (Conaty et al., 2018). Ensuring the future viability of the Australian cotton industry therefore commands the development of varieties that can maintain productivity in water-limited conditions, alongside continued improvements in management. The identification of water conservation mechanisms within existing cotton genotypes that can be selected in future breeding programs could be a viable strategy to improve drought tolerance.

Drought tolerance in plants is complex, controlled by a spectrum of physiological, anatomical, and biochemical mechanisms. As such, several adaptive strategies have evolved in plants to cope with drought stress. One water conservation mechanism that has been identified across a range of plant species is to reduce the rate of transpiration in response to drying soil. A general response to progressive soil drying across species and environments has been observed. Under these observations, transpiration rate is uninhibited until a threshold is reached, marking the initiation of stomatal closure, after which the transpiration rate decreases linearly in response to further soil drying (Devi et al., 2009; Ray and Sinclair, 1998). Soil water content may be expressed as a fraction of the total amount of water extracted for transpiration, i.e., the fraction of transpirable soil water (FTSW). Whilst early estimates of this transpiration breakpoint, or FTSW threshold for transpiration decline, were largely consistent between species (Lecoeur and Sinclair, 1996; Ray and Sinclair, 1997; Sadras and Milroy, 1996), more recent studies have identified significant genotypic variability in this threshold value (Choudhary et al., 2020; Devi and Reddy, 2020; Devi et al., 2009; De Souza et al., 2014; Wedegaertner et al., 2023). Devi et al. (2009) found significant variation between peanut genotypes in the FTSW value for transpiration decline from 0.22 to 0.71 FTSW. This variation was much greater than that previously reported for any other single species. Similarly, Gholipoor et al. (2013) found significant differences in FTSW thresholds of maize hybrids ranging from 0.33 to 0.60. Variation in the FTSW threshold has also been observed in other field crops and pasture species including sorghum (Gholipoor et al., 2012), field pea (Lecoeur and Sinclair, 1996), clover (Egan et al., 2021), potato (De Souza et al., 2014) and cotton (Devi and Reddy, 2020; Wedegaertner et al., 2023).

Genotypes that reduce transpiration when the FTSW is high may conserve soil water earlier in the season for use during subsequent dry periods. This water conservation strategy enables sustained crop productivity in prolonged water deficit conditions relative to genotypes with low FTSW thresholds that cannot conserve water for later use (Gholipoor et al., 2012; Ray and Sinclair, 1997). This reduces the risk of extreme dehydration between potential rainfall events and allows the crop to use stored water later in the season under a terminal drought scenario. The favourable performance of these genotypes in water-limited conditions therefore make these cultivars valuable breeding material in efforts to improve yields in specific water-limited environments, such as partially irrigated and rainfed systems. The difficulty around assessing the ability of plants to grow and develop in water limited environments revolves around traits associated with drought tolerance being correlated with plant survival. Since production goals are associated predominantly with lint yield and quality, relying on survival methods to overcome a lack of water availability will result in yield losses due to the lack of growth and development (Devi and Reddy, 2020; Sinclair, 2017b). Accordingly, there has been shift from a focus on specific traits, to a compilation of traits that allow a specific response, particularly soil moisture conservation.

Whilst commercial varieties targeting this trait have been released for both maize and sorghum, there is little research investigating variability in the FTSW threshold of cotton genotypes, particularly in Australia. The modern Australian cotton industry was initially based on varieties from the USA; however, domestic breeding efforts have led to the development of varieties more suited to the Australian environment (Constable et al., 2001; Liu et al., 2013; Conaty et al., 2022), with growth and physiology differences between older and modern cultivars (Broughton et al., 2017). Therefore, it is important to assess the transpiration responses of germplasm in the Australian cotton breeding program. The objectives of our investigation were to screen Australian germplasm for variation in the transpiration response to progressive soil drying, and to understand any differences in total water use between genotypes. We test the hypotheses that (1) there is genotypic variability within six genotypes for transpiration response to a progressive soil water deficit; and (2) genotype dependent responses to progressive soil drying are evident from leaf-level physiology, by measurement of gas exchange parameters. The development of drought tolerant germplasm for the Australian cotton industry will be beneficial in the projected increasingly frequent limited water environments resulting from a changing climate, and expansion of the Australian cotton industry into marginal regions.

## 2 Materials and methods

### 2.1 Growth conditions

Three glasshouse dry-down experiments with six cotton genotypes were conducted at the Australian Cotton Research Institute (ACRI) near Narrabri, NSW. These genotypes included two CSIRO breeding lines (CSX2027 and CSX8521) and four diverse, commercially released varieties (RC 89, DeltaPEARL, CS50 and Sicot 746B3F). These cultivars were selected based on known varietal characteristics and observations under water stressed conditions (**Table 1**). The glasshouse was maintained at 32°C/20°C (day/night) and was under natural light conditions. 9L pots were filled with a 3:1 field soil:perlite mixture and planted with approximately eight seeds per pot. In each experiment, five replicate pots were sown. The field soil, obtained from local ACRI fields, is a grey vertosol (Australian soil classification) with a pH of 7.3-7.6, clay fraction percentage of 60-65% and low organic matter content (Weaver et al., 2005). Prior to planting, 10g of MULTIgrow^®^ basal fertiliser (13.1% N, 4.5% P, 7.2% K, 15.4% S and 2.4% Ca) (Incitec Pivot Fertilisers, Melbourne, Australia) was added to the soil surface of each plot and dissolved with hand-held irrigation. Cotton seeds and the soil surface of each pot was covered with moist sand (~ 20 mm) and all soils were kept saturated via hand-held irrigation. This methodology has been identified to ensure uniform emergence of cotton in glasshouse studies using local field soils.

**Table 1:** Adapted from Broughton and Conaty (2022).

Genotype	Origin	Release year	Target environment reason for inclusion	Reference
CSX2027	CSIRO, Narrabri AU	n/a	Rainfed, limited water conservation in resp. to VPD	Broughton and Conaty (2022)
CSX8521	CSIRO, Narrabri AU	n/a	Rainfed, high irrigated yield, limited water conservation in resp. to VPD	Broughton and Conaty (2022)
CS 50	CSIRO, Narrabri AU	1992	Poor agronomic WUE, limited water conservation in resp. to VPD	Reid (1992) Broughton and Conaty (2022)
RC-89 (Syn. Surabhi)	Rasi Seeds, Attur India	1997	Diverse germplasm (India), observed drought tolerance in AU	
DeltaPEARL	Deltapine Australia Pty. Ltd., Goondiwindi AU	1999	Strong water conservation in resp. to VPD	Leske (2000) Broughton and Conaty (2022)
Sicot 746B3F	CSIRO, Narrabri AU	2016	Commercial irrigated Australian variety	Stiller (2017)

n/a = In these years, the breeding lines were not commercially released as a variety.

Experiment 1 was planted on 17 Jul. 2020; Experiment 2 on 4 Nov. 2020; and Experiment 3 on 21 Oct. 2021. At 20 days after planting (DAP) for all experiments, plants were thinned to one plant per pot and staked. Daily irrigation was then provided, with approximately 1200 mm of water supplied over a 15-minute period from 0900 hours via drip irrigators to saucers underneath pots.

Each experiment was set up as a randomised complete block design, with five replications. Factors constituted genotype and water treatment. Just prior to first square (development of the first floral buds; 45, 26 and 27 DAP for Experiments 1, 2 and 3, respectively), plants were blocked by size and divided into two water treatments; well-watered (control) and progressive water deficit. All pots were watered to field capacity. The surface of each pot was covered with aluminium foil to prevent soil evaporation before the pots were weighed to determine the initial pot weight. Well-watered plants were watered each morning (~0800 hrs) to the initial pot weight recorded on the first day of weighing. Water deficit plants were exposed to progressive soil drying (reduction in the FTSW) by imposing a daily water deficit of 65% of the previous day’s water use. This enabled extension of the drying cycle over several weeks to represent field drying conditions more accurately. The weight of each pot was recorded each morning, and the amount of water transpired each day was calculated as the change in pot weight between successive days. The experiments ran for a period of 34, 22 and 20 d for experiments 1, 2 and 3, respectively (79, 48 and 47 DAP, respectively). The experiment was concluded when the average ratio of water use from the water deficit and well-watered plants was< 0.1.

### 2.2 Leaf gas exchange

Leaf level gas exchange measurements were conducted on four occasions throughout the experimental period of Experiments 2 and 3. These measurements occurred at the initial pot weight (26 and 27 DAP for Experiments 2 and 3, respectively), at transpiration ratios of approximately 0.7 (36 and 37 DAP for Experiments 2 and 3, respectively) and 0.4 (40 and 41 DAP for Experiments 2 and 3, respectively), and on the final day of the experiment when the transpiration ratio was approximately 0.1 (47 and 48 DAP for Experiments 2 and 3, respectively). A portable open gas exchange system (LI-6400 XT and LI-6800, LI-COR Biosciences with the LI-6800 used on one to two replicates each sampling time due to machine availability) was used to measure photosynthetic rate, stomatal conductance and transpiration on one leaf per pot of a recently fully expanded leaf. There were no significant differences between replicates from the different IRGA models (P> 0.05). Measurements were conducted at mid-day growth temperature (32°C) and photosynthetic saturating light (photosynthetic photon flux density of 1800 µmol m^-2^ s^-1^). The CO_2_ concentration was maintained at 410 µL L^-1^ and vapour pressure deficit (VPD) of the air inside the lead chamber was maintained within 1.5-2.0 kPa. Gas exchange measurements were taken between approximately 0900 hours and 1400 hours (Australian Eastern Daylight Time). Genotypes and water treatments in each replicate were randomised to minimise the effects of the wide physiology measurement period. Furthermore, leaves were stabilised for at least two minutes to equilibrate before the measurement was recorded.

### 2.3 Plant growth measurements

At the end of the experiment water-stressed plants were saturated to ensure leaves were rehydrated. This rehydration process facilitated the measurement of leaf area which was conducted the following day. All plants were harvested and the number of leaves for each plant was measured. Total leaf area of each plant was measured using a portable leaf area meter (LI-3100A, LI-COR Biosciences). However, as leaf area measurements are destructive and because daily normalized transpiration rates needed to be calculated throughout the experiment period, the relationship between leaf number and leaf area at the end of the experiment was used to estimate the daily leaf area (see **Supplementary Material S1**). To develop this relationship the total leaf number of each plant was measured once each week from the initiation of the dry-down cycle in each experiment. This was used to estimate daily leaf number through a regression between measured leaf number and day. At the conclusion of the experiment the leaf area of a randomised sample of 20, 40 and 60 leaves, and the total leaf area of each plant, were measured to generate a leaf number vs. leaf area calibration curve for each genotype. A regression line was fit to the leaf area and leaf number data and used to estimate leaf area per plant per day (**Supplementary Material S2**). This daily leaf area measurement was used to calculate normalised daily transpiration rate (as below).

### 2.4 Calculations

Daily transpiration was normalised by the estimated leaf area (m^2^) of the plant on each day to account for variation in transpiration associated with plant size. Leaf areas were estimated using a calibration curve derived from the measured leaf number throughout the experiment and the leaf area measurements at the final harvest. The normalised daily transpiration (NDT) of each plant was calculated using the following formula from Devi and Reddy (2020):


$$\text{NDT}\;=\;\frac{\left(Difference\;in\;pot\;weight\;between\;sucessive\;days\right)}{\left(Estimated\;Leaf\;Area\right)}$$

The transpiration ratio was calculated using a paired approach. Each water deficit plant was paired with a corresponding well-watered plant by sorting the two sets of treatment data from lowest to highest NDT per pot, per genotype. This method was used to ensure plants that were most similar were paired. The transpiration ratio was then calculated using the following formula from Devi and Reddy (2020):


$$\text{Transpiration}\;\text{Ratio}=\;\frac{\left(NDT\;of\;water\;deficit\;plant\right)}{\left(NDT\;of\;well-watered\;plant\right)}$$

The total FTSW was calculated as the difference between the initial and final pot weight on the first and last day of each experiment, respectively. The FTSW on each day was calculated using the following formula from Devi and Reddy (2020):


$$\begin{array}{c} \text{Fraction}\;\text{transpirable}\;\text{soil}\;\text{water}\;\left(\text{FTSW}\right)\\ =\;\frac{\left(Daily\;pot\;weight-Final\;pot\;weight\right)}{\left(Initial\;pot\;weight-Final\;pot\;weight\right)} \end{array}$$

### 2.5 Statistical analyses

The daily transpiration ratio was plotted against FTSW for each replicate of each genotype. Two-segment linear regression was performed in Genstat version 19 (VSN International Ltd), where the FTSW value at the intersection of the two lines was extracted as the FTSW threshold of each replicate (see S3 for regression breakpoints and slopes for each replicate of each genotype). Analysis of variance (ANOVA) was used to assess variation in the FTSW threshold for transpiration decline between the varieties, where Fisher’s protected LSD was used to compare means of the 6 genotypes at P< 0.05.

To compare differences in gas exchange parameters between varieties and water treatments, stomatal conductance, transpiration and photosynthetic rate were determined for each plant on each day of measurement. Data were analysed using ANOVA, with each date and gas exchange parameter analysed separately. Both the main effects of water treatment and genotype were tested. No transformation of the data were required. Differences between the means of each water treatment and genotype for the various gas exchange parameters were compared using Tukey’s Kramer test (P< 0.05).

## 3 Results

### 3.1 FTSW threshold

Variation in both the FTSW threshold and rate of transpiration decline among the six genotypes were detected. The genotype CS 50 had the highest mean FTSW threshold values (FTSW = 0.354), which was 154% higher than that of RC-89 (the genotype tested with the lowest FTSW). The rate of transpiration decline was 45% faster for RC-89 compared with CSX2027 (**Table 2**). All genotypes were well characterised by the two-segment linear regression, displaying a clear breakpoint where transpiration decline was initiated, and for simplicity genotype FTSW means across experiments are presented (**Table 2**). Although significant experiment (p<0.001) and experiment-by-genotype interactions (p<0.001) were observed, the variance ratio of the experiment-by-genotype interaction explained<9% of the variability in the data, whereas the independent variables genotype and experiment accounted for 30% and 45% of the variance in the data, respectively. In each experiment CS 50 and CSX2027 consistently displayed the highest FTSW threshold. However, the experiment-by-genotype interaction could be observed in the remaining four genotypes which displayed some rank changes in FTSW thresholds, where in Experiment 1 DeltaPEARL displayed the lowest FTSW threshold, while RC-89 and Sicot 746B3F displayed the lowest FTSW thresholds in Experiments 2 and 3, respectively.

**Table 2:** The Fraction of transpirable soil water (FTSW) threshold and slope of the declining transpiration rate of 6 cotton genotypes.

Genotype	FTSW Threshold (Mean ± SE)	Slope (Mean ± SE)
CS 50	0.354 ± 0.04 c	3.591 ± 0.42 b
CSX2027	0.305 ± 0.03 b	2.967 ± 0.21 a
CSX8521	0.255 ± 0.03 a	3.425 ± 0.40 ab
DeltaPEARL	0.248 ± 0.04 a	3.800 ± 0.45 bc
Sicot 746B3F	0.233 ± 0.03 a	3.460 ± 0.40 ab
RC-89	0.230 ± 0.04 a	4.295 ± 0.48 c

Values with the same letters are not significantly different from each other at P< 0.05.

Numbers are the mean ± standard error of the mean from Experiments 1, 2, and 3.

### 3.2 Rate of productivity over the drying cycle

**Figure 1:**
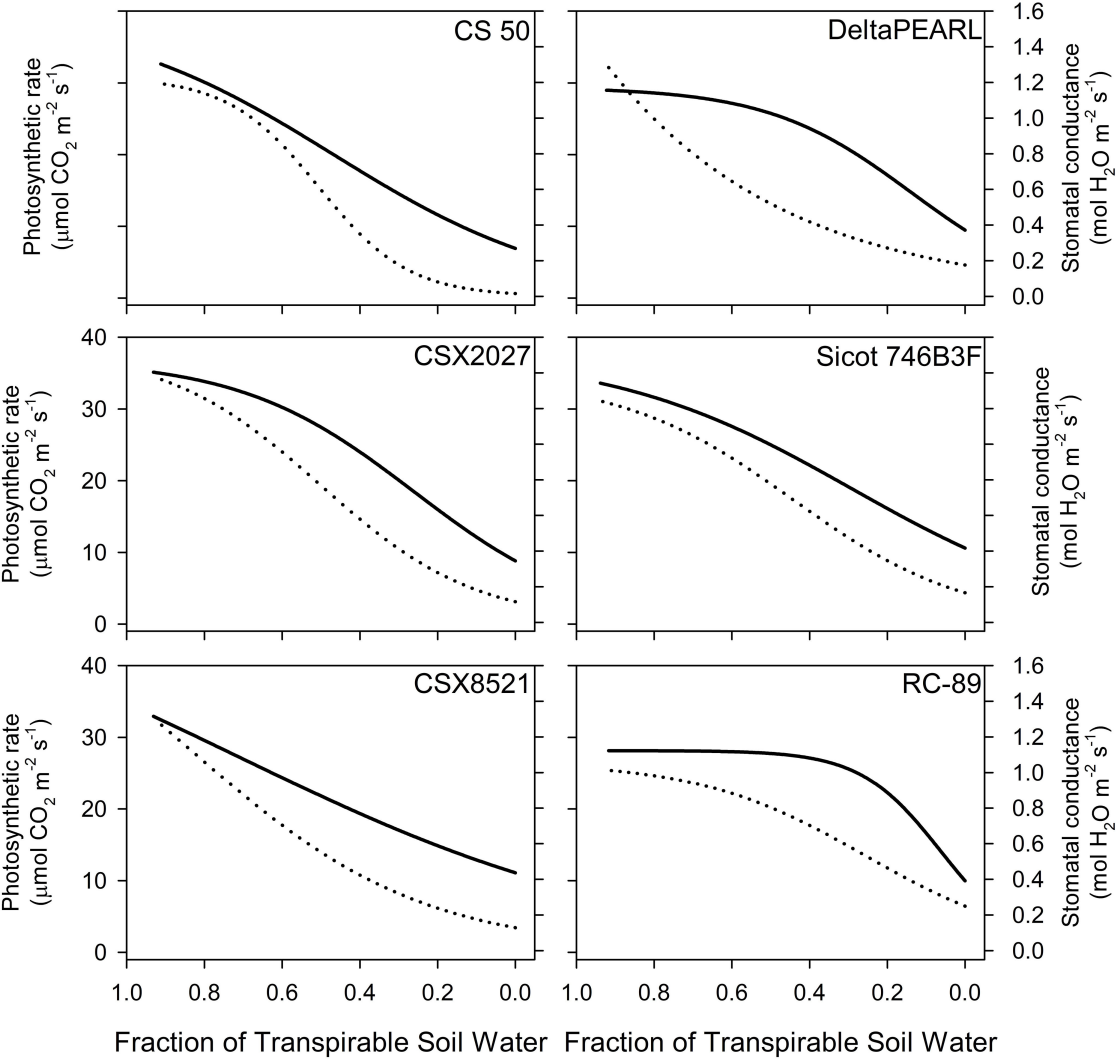
Photosynthetic (solid line) and stomatal (dotted line) response to the fraction of transpirable soil water (FTSW) for six cotton genotypes measured in Experiments 2 and 3, n=40. Fitted response curves and statistics are shown in **Supplementary Material (S4)**.

**Figure 2:**
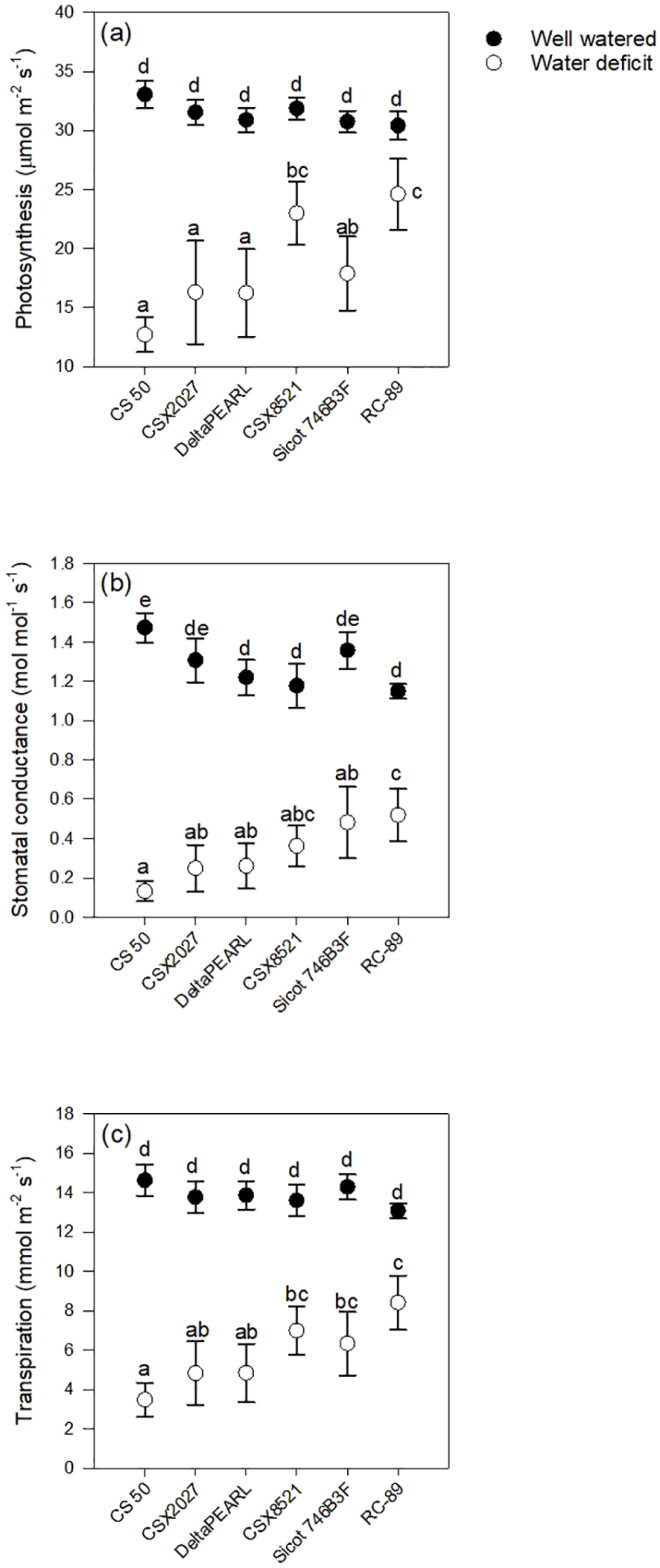
Interactive effect of water treatment and genotype on **(A)** photosynthesis, **(B)** stomatal conductance, and **(C)** transpiration at the timepoint when water-deficit plants reached FTSW= 0.4 across Experiments 2 and 3. Values represent mean ±SE. Letters represent significant difference at P< 0.05 using Fisher’s LSD.

Genotypes with high FTSW thresholds, such as CS 50 and CSX2027 had greater initial photosynthetic rates, stomatal conductance and transpiration than that of RC-89, with a lower FTSW threshold (**Figure 1**, **Table 3**). Both CS 50 and CSX2027 displayed a more rapid decline in photosynthetic rate and stomatal conductance that commenced earlier in the drying cycle than cultivars with lower FTSW thresholds, like RC-89. In contrast, RC-89 maintained a consistent photosynthetic rate for a greater extent of the soil drying cycle, until ~0.1 FTSW, after which a rapid decline occurred (**Figure 1**). A significant genotype-by-water treatment interaction for photosynthesis, stomatal conductance, and transpiration rates occurred when at the FTSW= 0.4 gas exchange measurements (**Figure 2**). When FTSW= 0.4, photosynthesis, stomatal conductance, and transpiration rates of water-stressed CS 50 plants were 48%, 75% and 59%, respectively, lower than water-stressed RC-89 plants.

**Table 3:** ANOVA table for the effects of water treatment and genotype on the physiology of cotton at four FTSW time points for Experiments 2 and 3.

		FTSW 1	FTSW 0.7	FTSW 0.4	FTSW 0.1
Photosynthesis	Water Trt	0.547	**0.001**	**0.001**	**0.001**
	Genotype	**0.001**	0.101	0.084	**0.023**
	Water x Genotype	0.210	0.328	**0.010**	0.896
Stomatal Conductance	Water Trt	0.303	**0.001**	**0.001**	**0.001**
	Genotype	**0.001**	**0.035**	0.406	0.819
	Water x Genotype	0.350	0.105	**0.004**	0.933
Transpiration	Water Trt	0.350	**0.001**	**0.001**	**0.001**
	Genotype	**0.019**	0.109	0.254	0.261
	Water x Genotype	0.490	0.330	**0.006**	0.949

Values in bold represent statistical significance at P< 0.05.

## 4 Discussion

Genotypic variation in the transpiration response to progressive soil drying was observed at both the whole plant and leaf level among the six cotton genotypes. This result supports our first hypothesis, that there is genotypic variability within the six genotypes tested for transpiration response to a progressive soil water deficit. Differences in the FTSW thresholds were found, ranging from 0.23 to 0.35. Genotypes CSX2027 and CS 50 had the highest FTSW thresholds for transpiration decline, which were greater than that of RC-89. Gas exchange parameters also varied between varieties throughout the drying cycle, with differences in stomatal conductance and photosynthetic rate attributable to genotypic differences in the initiation of stomatal closure. This result supports our second hypothesis that genotype dependent responses to progressive soil drying will be evident, with respect to leaf-level physiology.

### 4.1 Variation in the FSTW threshold

Genotypic variability in the transpiration response to soil drying conditions has been observed across a range of plant species (De Souza et al., 2014; Devi and Reddy, 2020; Devi et al., 2009; Gholipoor et al., 2012; Gholipoor et al., 2013; Lecoeur and Sinclair, 1996; Wedegaertner et al., 2023). Devi and Reddy (2020) found significant variation in the FTSW threshold of 15 cotton varieties, with the FTSW threshold ranging from 0.35 to 0.60. These values are higher, and range more broadly than the FSTW thresholds for the six cotton genotypes investigated in our study, which ranged from 0.23 to 0.35. Whilst Devi and Reddy (2020) found the FSTW threshold of CS 50 to be 0.53, here it was found to be 0.35. These inconsistencies may be attributable to differences in experimental methodology and conditions including day length, glasshouse temperature and soil type. For example, our study used pots with approximately three times the volume (9L vs 3L pots), which have a much larger water holding and buffering capacity during the dry down cycle. In addition, the dry down cycle was intentionally extended for a longer period (up to 34 d vs 16 d). These two aspects of our study were designed to mimic field conditions where plants have access to significantly more water than under glasshouse conditions. However, it must be noted that this experimental design may have primed plants to acclimate to the drought stress conditions (Flexas et al., 2006). Despite these differences in the experimental design and the resulting threshold values, in both studies, CS 50 displayed a significantly higher FSTW threshold than multiple other varieties. This suggests that the physiological response of CS 50 to a drying soil profile may provide improved performance relative to the other five genotypes tested in our study. Interestingly, a recent study by Wedegaertner et al. (2023) which was also conducted in cotton, identified a range of FTSW thresholds much more similar to our study, 0.29 to 0.39.

### 4.2 Higher FTSW thresholds are more favourable under prolonged soil water deficit

Initiating stomatal closure at a high FTSW threshold enables water conservation to commence earlier in the season, meaning there is more water available to sustain productivity over an extended dry period (Ray and Sinclair, 1997; Sinclair, 2017b). In contrast, genotypes that limit transpiration at low FTSW will have less water stored in the soil once the FTSW threshold is reached. These water resources will therefore be exhausted more rapidly, increasing the likelihood of a FTSW being reached that will significantly impact productivity. In their simulated model study, Sinclair and Muchow (2001) showed that when stomatal closure was delayed, a lethal FTSW occurred in 16 out of 20 years with high interannual variability in rainfall in Columbia, MO, USA. Whilst traditionally, high yields and fibre quality have been achieved in Australia with more than 85% of cotton production systems being irrigated, reductions in water availability in the near future will necessitate further expansion of the cotton industry into partially irrigated or rainfed systems. Consequently, the industry will potentially benefit from genotypes with high FTSW thresholds. These genotypes may provide improved productivity under prolonged water-deficit conditions, where existing varieties bred in a fully irrigated target environment may not have the adaptation required in these future limited water environments. Results from this study suggest that CS 50 and CSX2027, which had the highest FTSW thresholds, may therefore provide valuable breeding stock for improving performance in systems with extended dry periods.

### 4.3 Lower FTSW thresholds are more favourable under intermittent water deficit

The consequence of early stomatal closure in genotypes with high FSTW thresholds is a reduction in CO_2_ assimilation due to limited gas exchange (Sinclair, 2017a). Therefore, there is a trade-off between sustained physiological activity during prolonged dry periods and reduced photosynthetic activity. Genotypes with low FTSW thresholds, such as Sicot 746B3F and RC-89, are therefore more advantageous under fully irrigated conditions or when the drought is short and intermittent, as a higher level of productivity is maintained for a greater extent of the soil drying cycle. Premature stomatal closure will result in lost productivity for genotypes with high FTSW thresholds when soil water is replenished shortly after transpiration decline is initiated. Thus, the relative advantage of high or low FTSW thresholds is dependent on the type of water deficit scenario that is endured. Fuentealba et al. (2016) therefore suggest a regional approach to improving performance under water deficit, where genotypes are selected according to the common water deficit scenario of a region.

### 4.4 Productivity during the soil drying cycle

RC-89 displayed a conservative approach to productivity in the well-watered phase, with a photosynthetic rate that was ~20% less than that of CSX2027 on the first day of the experiment, before soil drying commenced (**Figure 1**, **Supplementary Material S2**). This inherently low productivity in the initial phase of soil drying allows some conservation of water that can be utilised later in the soil drying cycle. This is evident in **Figure 1**, where photosynthetic rate is relatively lower for RC-89 at the start of the drying cycle, but as drying continues these rates are maintained until FTSW is equal to ~0.3, after which a rapid decline occurs. Contrastingly, for genotypes with inherently high photosynthesis (and stomatal conductance), particularly CSX8521 and CS 50, the rate of productivity starts declining earlier in the drying cycle, with a more gradual decline at lower FTSWs (i.e.,<0.3). DeltaPEARL, CSX2027 and Sicot 746B3F (to a lesser degree) represent more intermediate productivity curves where photosynthesis rates are maintained for the initial drying phase (FTSW>0.75), and then gradually decline. Similarly, these observations were also evident from gas exchange measurements taken 40 DAP, where the photosynthetic rate of RC-89 (31 µmol CO_2_ m^-2^ s^-1^) was approximately two and two and a half times higher than that of CSX2027 and CS 50, respectively, (i.e., 16 and 13 µmol CO_2_ m^-2^ s^-1^) under water deficit. These unique mechanisms for water conservation were also observed by Gholipoor et al. (2012), who found sorghum genotypes displayed one of two distinct traits; a high FTSW threshold to conserve water early in the drying cycle, or a lower FTSW threshold that was coupled with lower inherent transpiration rates under well-watered conditions. This provides further evidence that the relative advantage of a high or low transpiration breakpoint depends on the water deficit scenario. RC-89 will be higher yielding in short-term water deficits where soil water is replenished prior to the rapid crash in productivity. Contrastingly, physiological activity can be maintained for longer under a prolonged water deficit when the decline in stomatal conductance and photosynthesis is more gradual. These results are presented in the context of a genotype’s relative yield performance across water environments. However, it is of vital importance that these results are also interpreted in the context of a genotype’s yield potential. Elite and more modern varieties such as Sicot 746B3F have a much greater yield potential than older and locally non-adapted varieties such as RC-89 which has significantly reduced growth and partitioning potential, rather than just the impact of physiological breakpoints.

## 5 Conclusion

This investigation showed genotypic variation among six cotton genotypes for their FTSW thresholds for transpiration decline. Although the range of variance in the FSTW threshold was relatively small, these results provide further evidence that significant genotypic variation in the transpiration response to progressive soil drying exists in cotton. These genotype dependent responses to soil drying were also evident from leaf gas exchange measurements, with reductions in stomatal conductance and photosynthetic rate coinciding with the FTSW threshold of each genotype. A trade-off between reduced photosynthetic activity and sustained physiological activity over prolonged water-deficit was found. Therefore, genotypes with low FTSW thresholds may provide improved performance in short water-deficit scenarios, owing to a higher level of productivity over a greater extent of the soil drying cycle. Further investigations are necessary before commercial varieties targeting this trait can be developed. The transpiration responses found here were observed under highly controlled glasshouse conditions, therefore, further studies investigating how these genotypic differences affect productivity in the field are required. Quantifying yield differences among varieties based on their FTSW thresholds is critical to understand which genotypes will provide improved performance under water deficit scenarios. Additionally, a larger genetic pool should be investigated as the range of FTSW values observed here was relatively small compared to other species. These data suggest that the TR_Lim_ (limited transpiration) FTSW trait can be identified, which may provide benefit for selecting more drought tolerant genotypes to integrate into the cotton breeding program, particularly for use in rainfed and partially irrigated cotton systems. However, detailed physiological studies such as this cannot be scaled to result in direct application to breeding programs. Therefore, additional studies must also be conducted to identify ways that this knowledge can be applied in a crop improvement context. One avenue that this may be achieved is through the integration of this physiological understanding in genomic prediction models through the weighting of markers associated genes and transcription factors associated with the measured physiological responses.

## Data Availability

The raw data supporting the conclusions of this article will be made available by the authors, without undue reservation.

## References

[B1] Broughton K. J. Bange M. P. Duursma R. A. Payton P. Smith R. A. Tan D. K. Y.. (2017). The effect of elevated atmospheric [CO2] and increased temperatures on an older and modern cotton cultivar. Funct. Plant Biol. 44, 1207–1218. doi: 10.1071/FP17165 32480645

[B2] Broughton K. J. Conaty W. C. (2022). Understanding and exploiting transpiration response to vapor pressure deficit for water limited environments. Front. Plant Sci. 13, 893994. doi: 10.3389/fpls.2022.893994 PMC9127727 35620701

[B3] Choudhary S. Guha A. Kholova J. Pandravada A. Messina C. D. Cooper M.. (2020). Maize, sorghum, and pearl millet have highly contrasting species strategies to adapt to water stress and climate change-like conditions. Plant Sci. 295. doi: 10.1016/j.plantsci.2019.110297 32534623

[B4] Conaty W. C. Broughton K. J. Egan L. M. Li X. Li Z. Liu S.. (2022). Cotton breeding in Australia: meeting the challenges of the 21st century. Front. Plant Sci. 13. doi: 10.3389/fpls.2022.904131 PMC9136452 35646011

[B5] Conaty W. C. Johnston D. B. Thompson A. J. E. Liu S. M. Stiller W. N. Constable G. A. (2018). Use of a managed stress environment in breeding cotton for a variable rainfall environment. Field Crops Res. 221, 265–276. doi: 10.1016/j.fcr.2017.10.012

[B6] Constable G. A. Thomson J. J. Reid P. E. (2001). “Approaches utilized in breeding and development of cotton cultivars in Australia,” in Genetic improvement of cotton. Eds. Jenkins J. N. Saha S. (Science Publishers, Enfield).

[B7] De Souza A. T. Streck N. A. Heldwein A. B. Bisognin D. A. Winck J. E. M. Da Rocha T. S. M.. (2014). Transpiration and leaf growth of potato clones in response to soil water deficit. Scientia Agricola 71, 96–104. doi: 10.1590/S0103-90162014000200002

[B8] Devi M. J. Reddy V. (2020). Cotton genotypic variability for transpiration decrease with progressive soil drying. Agronomy 10, 1290. doi: 10.3390/agronomy10091290

[B9] Devi M. J. Sinclair T. R. Vadez V. Krishnamurthy L. (2009). Peanut genotypic variation in transpiration efficiency and decreased transpiration during progressive soil drying. Field Crops Res. 114, 280–285. doi: 10.1016/j.fcr.2009.08.012

[B10] Egan L. Hofmann R. Nichols S. Hadipurnomo J. Hoyos-Villegas V. (2021). Transpiration rate of white clover (Trifolium repens L.) cultivars in drying soil. Front. Plant Sci. 12. doi: 10.3389/fpls.2021.595030 PMC8010265 33815432

[B11] Flexas J. Bota J. Galmés J. Medrano H. RIBAS-Carbó M. (2006). Keeping a positive carbon balance under adverse conditions:: responses of photosynthesis and respiration to water stress. Physiologia Plantarum 127, 343–352. doi: 10.1111/j.1399-3054.2006.00621.x

[B12] Fuentealba M. P. Zhang J. Kenworthy K. Erickson J. Kruse J. Trenholm L. (2016). Transpiration responses of warm-season turfgrass in relation to progressive soil drying. Scientia Hortic. 198, 249–253. doi: 10.1016/j.scienta.2015.11.042

[B13] Gholipoor M. Sinclair T. R. Prasad P. V. V. (2012). Genotypic variation within sorghum for transpiration response to drying soil. Plant Soil 357, 35–40. doi: 10.1007/s11104-012-1140-8

[B14] Gholipoor M. Sinclair T. R. Raza M. A. S. Löffler C. Cooper M. Messina C. D. (2013). Maize hybrid variability for transpiration decrease with progressive soil drying. J. Agron. Crop Sci. 199, 23–29. doi: 10.1111/j.1439-037X.2012.00530.x

[B15] Hearn A. B. (1979). Water relationships in cotton. Outlook Agric. 10, 159–166. doi: 10.1177/003072708001000402

[B16] Hearn A. B. (1994). “The principles of cotton water relations and their application in management,” in Challenging the Future- Proceedings of the World Cotton Research Conference 1. Eds. Constable G. A. Forrester N. W. (CSIRO, Brisbane, Australia).

[B17] Lecoeur J. Sinclair T. R. (1996). Field pea transpiration and leaf growth in response to soil water deficits. Crop Sci. 36, 331–335. doi: 10.2135/cropsci1996.0011183X003600020020x

[B18] Leske R. (2000). DeltaPEARL. Plant Varieties J. 10, 18. Available at: https://www.ipaustralia.gov.au/tools-and-research/professional-resources/ip-rights-journals/~/-/media/Project/IPA/IPAustralia/PDF/PBR-Journals/Volume-10-Number-3.pdf?rev=a67bfa72116647afa9d007fb7d96549c

[B19] Liu S. M. Constable G. A. Reid P. E. Stiller W. N. Cullis B. R. (2013). The interaction between breeding and crop management in improved cotton yield. Field Crops Res. 148, 49–60. doi: 10.1016/j.fcr.2013.04.006

[B20] Pimentel D. Berger B. Filiberto D. Newton M. Wolfe B. Karabinakis E.. (2004). Water resources: Agricultural and environmental issues. Bioscience 54, 909–918. doi: 10.1641/0006-3568(2004)054[0909:WRAAEI]2.0.CO;2

[B21] Ray J. D. Sinclair T. R. (1997). Stomatal closure of maize hybrids in response to drying soil. Crop Sci. 37, 803–807. doi: 10.2135/cropsci1997.0011183X003700030018x

[B22] Ray J. D. Sinclair T. R. (1998). The effect of pot size on growth and transpiration of maize and soybean during water deficit stress. J. Exp. Bot. 49, 1381–1386. doi: 10.1093/jxb/49.325.1381

[B23] Reid P. (1992). CS 50. Plant Varieties J. 5, 12. Available online at: https://www.ipaustralia.gov.au/tools-and-research/professional-resources/ip-rights-journals/~/-/media/Project/IPA/IPAustralia/PDF/PBR-Journals/Volume-5-Number-2.pdf?rev=d3760397da8242f18dddac7a222b7026

[B24] Roth G. Harris G. Gillies M. Montgomery J. Wigginton D. (2013). Water-use efficiency and productivity trends in Australian irrigated cotton: a review. Crop Pasture Sci. 64, 1033–1048. doi: 10.1071/CP13315

[B25] Sadras V. O. Milroy S. P. (1996). Soil-water thresholds for the responses of leaf expansion and gas exchange: A review. Field Crops Res. 47, 253–266. doi: 10.1016/0378-4290(96)00014-7

[B26] Sinclair T. R. (2017a). “Early partial stomata closure with soil drying,” in Water-Conservation Traits to Increase Crop Yields in Water-Deficit Environments: Case Studies. Ed. Sinclair T. R. (Switzerland: Springer International Publishing Ag, Cham).

[B27] Sinclair T. R. (2017b). “Water-conservation traits to increase crop yields in waterdeficit environments case studies introduction, “ in Water-Conservation Traits toIncrease Crop Yields in Water-Deficit Environments: Case Studies. Ed. Sinclair T. R..

[B28] Sinclair T. R. Muchow R. C. (2001). System analysis of plant traits to increase grain yield on limited water supplies. Agron. J. 93, 263–270. doi: 10.2134/agronj2001.932263x

[B29] Stiller W. (2017). Sicot 746B3F. Plant Varieties J. 29, 128–132. Available online at: https://www.ipaustralia.gov.au/tools-and-research/professional-resources/ip-rights-journals/~/-/media/Project/IPA/IPAustralia/PDF/PBR-Journals/Volume-29-Number-4.pdf?rev=8f18d8fd44bc4f6fac066bf228ee42ec

[B30] Weaver T. B. Hulugalle N. R. Ghadiri H. (2005). Comparing deep drainage estimated with transient and steady state assumptions in irrigated vertisols. Irrigation Sci. 23, 183–191. doi: 10.1007/s00271-005-0106-5

[B31] Wedegaertner K. Shekoofa A. Sheldon K. Simón J. Raper T. B. (2023). Screening cotton cultivars under induced water-deficit stress in controlled environments and field settings: expression of drought tolerance traits. J. Crop Improvement 37, 395–416. doi: 10.1080/15427528.2022.2098217

[B32] Williams A. Mushtaq S. Kouadio L. Power B. Marcussen T. Mcrae D.. (2018). An investigation of farm-scale adaptation options for cotton production in the face of future climate change and water allocation policies in southern Queensland, Australia. Agric. Water Manage. 196, 124–132. doi: 10.1016/j.agwat.2017.10.026

