# Supplementary material for: Genotypic variability in cotton's transpiration response under progressive soil drying
Source: Front Plant Sci. 2024 Dec 4;15:1451993. doi: 10.3389/fpls.2024.1451993 (PMC11652182; doi:10.3389/fpls.2024.1451993)
Supplement: Supplementary file 1 [file DataSheet1.pdf]

Supplementary Material

Cotton genotypic variability in the transpiration response to progressive soil drying

Katrina J. Broughton*[1†], Eleanor L. Bate[1,2], Corey W. Cutler[1,3], Christopher N. Allen[1], Alan J. E. Thompson[1], Warren C. Conaty[1†]

*** Correspondence:** Katie Broughton: katie.broughton@csiro.au

1 Supplementary Tables

S1: Equations of regressions between the number of leaves and leaf area for each genotype of each water treatment in each experiment.

Experiment	Genotype	Well-Watered	R^2	Water Deficit	R^2
1	CS 50	y = 68.808x - 268.39	0.80	y = 48.253x + 522.13	0.76
	CSX2027	y = 65.596x - 604.3	0.91	y = 54.592x - 172.83	0.85
	CSX8521	y = 48.956x + 404.46	0.95	y = 48.938x + 557.44	0.96
	DeltaPEARL	y = 67.06x + 252.91	0.94	y = 74.555x - 278.36	0.90
	RC-89	y = 38.905x + 263.82	0.96	y = 34.308x + 369.85	0.85
	Sicot 746B3F	y = 50.45x + 255.8	0.95	y = 43.267x + 403.3	0.88
2	CS 50	y = 79.447x - 218.47	0.96	y = 65.195x - 112.33	0.92
	CSX2027	y = 66.8x - 190.87	0.84	y = 49.299x + 105.69	0.87
	CSX8521	y = 65.275x - 177.95	0.91	y = 52.973x + 12.651	0.88
	DeltaPEARL	y = 78.862x - 90.742	0.95	y = 54.263x + 125.75	0.90
	RC-89	y = 77.869x - 350.65	0.88	y = 57.071x - 135.68	0.91
	Sicot 746B3F	y = 69.239x + 98.434	0.98	y = 57.174x + 40.64	0.87
3	CS 50	y = 54.904x - 347.01	0.89	y = 34.498x + 94.085	0.72
	CSX2027	y = 56.32x - 389.91	0.89	y = 33.32x + 144.81	0.77
	CSX8521	y = 54.745x - 399.82	0.93	y = 46.896x - 268.58	0.85
	DeltaPEARL	y = 53.831x - 324.97	0.87	y = 39.812x - 11.595	0.78
	RC-89	y = 51.678x - 261.71	0.88	y = 30.344x + 166.47	0.62
	Sicot 746B3F	y = 55.979x - 460.28	0.88	y = 39.725x - 107.98	0.94

S2: Equations of regressions between day of experiment and leaf area for each plant in each experiment.

Experiment	Genotype	Rep	Well-Watered	R^2	Water -Deficit	R^2
1	CS 50	1	y = 79.56x + 1853.5	0.88	$y = -4.7271x^2 + 248.84x + 1365.4$	0.99
		2	y = 290.97x + 1252.8	0.99	$y = -5.3753x^2 + 272.16x + 1209.7$	0.99
		3	y = 101.9x + 1425.9	0.92	$y = -3.5641x^2 + 191.73x + 967.78$	0.99
		4	y = 216.2x + 882.61	0.99	$y = -6.3139x^2 + 268.76x + 1055.7$	0.99
		5	y = 132.76x + 1047.4	0.97	$y = -7.4459x^2 + 311.6x + 1159.9$	0.96
	CSX2027	1	y = 172.6x + 937.64	0.99	$y = -3.2384x^2 + 213.11x + 821.05$	0.99
		2	y = 179.23x + 985.2	0.99	$y = -4.5064x^2 + 243.52x + 769.84$	0.99
		3	y = 190.93x + 1103.4	0.99	$y = -6.086x^2 + 269.05x + 828.07$	0.99
		4	y = 177.09x + 1784.6	0.96	$y = -2.9951x^2 + 221.57x + 923.12$	0.99
		5	y = 166.82x + 811.29	0.99	$y = -4.4535x^2 + 253.52x + 710.38$	0.99
	CSX8521	1	y = 161.01x + 2176.1	0.99	$y = -2.6927x^2 + 131.6x + 2330.4$	0.92
		2	y = 83.883x + 1398.9	0.97	$y = -2.8015x^2 + 166.4x + 1810.4$	0.99
		3	y = 129.27x + 1476.6	0.99	$y = -2.9779x^2 + 155.28x + 1371.1$	0.99
		4	y = 167.69x + 1790.5	0.99	$y = -4.1675x^2 + 204.08x + 1525.3$	0.99
		5	y = 103.87x + 1579.7	0.99	$y = -2.807x^2 + 148.4x + 1287.1$	0.99
	DeltaPEARL	1	y = 89.414x + 922.24	0.99	$y = -3.6493x^2 + 190.11x + 470.53$	0.96
		2	y = 155.67x + 514.52	0.99	$y = -2.0852x^2 + 127.91x + 533.28$	0.98
		3	y = 93.051x + 698.23	0.98	$y = -1.4279x^2 + 126.81x + 575.31$	0.99
		4	y = 137.72x + 384.93	0.99	$y = -2.7341x^2 + 174x + 485.15$	0.99
		5	y = 108.5x + 442.53	0.99	$y = -2.0676x^2 + 150.58x + 358.56$	0.99
	RC-89	1	y = 91.254x + 1203.1	0.99	$y = -2.9126x^2 + 146.24x + 814.7$	0.98
		2	y = 89.153x + 1260.5	0.99	$y = -3.5817x^2 + 161.94x + 1079$	0.99
		3	y = 102.85x + 1123.3	0.99	$y = -3.5554x^2 + 179.39x + 1122.6$	0.99
		4	y = 115.75x + 1301.4	0.99	$y = -1.998x^2 + 108.83x + 892.52$	0.96
		5	y = 63.305x + 1065.2	0.98	$y = -2.4481x^2 + 121.67x + 892.33$	0.98
	Sicot 746B3F	1	y = 93.42x + 1806.1	0.97	$y = -3.7551x^2 + 182.52x + 1686.6$	0.99
		2	y = 102.05x + 1405.7	0.99	$y = -1.7632x^2 + 114.89x + 648.81$	0.99
		3	y = 101.64x + 1458.3	0.98	$y = -3.7123x^2 + 193.82x + 1184.4$	0.99
		4	y = 119.55x + 1438.1	0.99	$y = -1.7486x^2 + 107.16x + 906.57$	0.99
		5	y = 102.55x + 1231.1	0.99	$y = -0.8452x^2 + 83.377x + 1072.9$	0.99
2	CS 50	1	y = 172.51x + 578.27	0.99	$y = -6.0801x^2 + 220.42x + 190.84$	0.99
		2	y = 200.89x + 589.62	0.95	$y = -10.134x^2 + 302.3x + 430.81$	0.99
		3	y = 220.18x + 427.32	0.99	$y = -10.134x^2 + 320.46x + 341.14$	0.99
		4	y = 206.56x + 862.01	0.96	$y = -11.35x^2 + 324.6x + 310.42$	0.98
		5	y = 171.38x + 253.67	0.99	$y = -5.6748x^2 + 189.54x + 487.47$	0.86

	CSX2027	1	y = 191.81x + 158.4	0.99	y = -2.7265x^2 + 158.14x + 121.32	0.99
		2	y = 118.33x + 151.72	0.99	y = -9.5429x^2 + 307.28x + 292.95	0.98
		3	y = 144.1x + 506.71	0.93	y = -8.8612x^2 + 299.24x + 86.554	0.99
		4	y = 194.67x + 576.38	0.99	y = -9.202x^2 + 323.3x + 226.49	0.99
		5	y = 191.81x + 893.2	0.95	y = -4.7714x^2 + 195.63x + 219.47	0.99
	CSX8521	1	y = 177.18x + 591.36	0.99	y = -11.323x^2 + 370.47x - 60.588	0.99
		2	y = 164.12x + 154.02	0.99	y = -8.6589x^2 + 284.95x + 244.21	0.95
		3	y = 189.3x + 566.19	0.97	y = -7.9929x^2 + 264.03x + 323.2	0.99
		4	y = 155.73x + 266.85	0.99	y = -8.6589x^2 + 298x + 257.26	0.98
		5	y = 207.02x + 607.22	0.99	y = -9.9911x^2 + 321.18x + 346.38	0.99
	DeltaPEARL	1	y = 201.74x + 421.68	0.99	y = -5.6039x^2 + 225.28x + 176.23	0.99
		2	y = 199.5x + 604.37	0.96	y = -8.4059x^2 + 268.43x + 1144	0.89
		3	y = 173.72x + 292.79	0.99	y = -3.2022x^2 + 226.08x + 173.03	0.99
		4	y = 243.21x + 121.31	0.99	y = -11.208x^2 + 336.24x + 494.53	0.96
		5	y = 244.33x + 441.86	0.99	y = -3.2022x^2 + 174.52x + 334.42	0.99
	RC-89	1	y = 268.77x - 160.64	0.98	y = -6.7993x^2 + 303.09x - 199.91	0.97
		2	y = 253.09x + 19.665	0.99	y = -6.7993x^2 + 291.89x + 46.462	0.99
		3	y = 283.33x - 269.27	0.99	y = -10.399x^2 + 328.77x + 103.34	0.96
		4	y = 260.93x - 325.26	0.95	y = -9.999x^2 + 320.69x + 20.865	0.99
		5	y = 291.17x + 561.69	0.99	y = -10.399x^2 + 301.89x - 18.731	0.99
	Sicot 746B3F	1	y = 166.12x + 265.91	0.99	y = -4.2632x^2 + 227.37x + 229.81	0.99
		2	y = 172.09x + 197.27	0.99	y = -5.3291x^2 + 244.92x + 147.18	0.99
		3	y = 167.12x + 480.78	0.99	y = -5.6843x^2 + 236.18x + 438.29	0.99
		4	y = 171.1x + 260.94	0.99	y = -6.3949x^2 + 262.47x + 99.359	0.95
		5	y = 170.1x + 603.13	0.96	y = -8.8818x^2 + 282.87x + 363.47	0.98
3	CS 50	1	y = 91.105x + 509.53	0.96	y = -0.3659x^2 + 61.443x + 517.82	0.92
		2	y = 74.087x + 940.13	0.97	y = -2.5613x^2 + 73.589x + 323.77	0.97
		3	y = 73.056x + 488.91	0.95	y = -2.5613x^2 + 92.497x + 374.65	0.97
		4	y = 87.151x + 636.74	0.94	y = -4.7566x^2 + 142.8x + 458.38	0.82
		5	y = 79.416x + 799.87	0.97	y = -2.1954x^2 + 82.623x + 383.38	0.97
	CSX2027	1	y = 104.42x	0.97	y = -2.4987x^2 + 88.039x + 583.25	0.91
		2	y = 88.406x + 590.38	0.96	y = -2.1233x^2 + 74.857x + 542.36	0.99
		3	y = 104.72x + 635.75	0.98	y = -3.8928x^2 + 120.99x + 581.51	0.94
		4	y = 96.117x + 637.36	0.99	y = -4.7228x^2 + 145.07x + 649.92	0.78
		5	y = 100.6x + 982.2	0.85	y = -1.9639x^2 + 67.601x + 482.97	0.77
	CSX8521	1	y = 83.876x + 372.91	0.97	y = -5.1123x^2 + 120.23x + 472.94	0.99
		2	y = 128.4x + 844.58	0.84	y = -4.3258x^2 + 113.04x + 661.13	0.95
		3	y = 87.016x + 439.42	0.99	y = -5.1122x^2 + 134.27x + 477.19	0.96

	4	y = 116.39x + 587.04	0.98	$y = -1.573x^2 + 80.139x + 801.67$	0.97
	5	y = 89.788x + 564.5	0.92	$y = -6.6853x^2 + 149x + 289.99$	0.99
DeltaPEARL	1	y = 84.183x + 624.05	0.98	$y = -2.2682x^2 + 81.458x + 595.12$	0.98
	2	y = 102.3x + 716.58	0.96	$y = -3.0243x^2 + 92.271x + 377.09$	0.99
	3	y = 98.924x + 629.55	0.95	$y = -2.2682x^2 + 77.906x + 655.33$	0.99
	4	y = 78.499x + 413.41	0.96	$y = -1.5121x^2 + 63.54x + 774.79$	0.99
	5	y = 83.65x + 562.95	0.98	$y = -3.0243x^2 + 95.823x + 475.66$	0.98
RC-89	1	y = 49.338x + 716.04	0.62	$y = -2.1156x^2 + 75.405x + 384.16$	0.84
	2	y = 111.1x + 357.41	0.95	$y = -6.2305x^2 + 157.61x + 320.42$	0.92
	3	y = 83.94x + 703.03	0.85	$y = -2.6067x^2 + 71.52x + 702.39$	0.50
	4	y = 120.15x + 454.55	0.96	$y = -2.2357x^2 + 57.724x + 732.9$	0.79
	5	y = 82.982x + 671.38	0.92	$y = -3.9764x^2 + 87.344x + 612.91$	0.80
Sicot 746B3F	1	y = 90.179x + 478.85	0.85	$y = -2.8792x^2 + 99.776x + 373.44$	0.74
	2	y = 75.717x + 595.83	0.72	$y = -2.95x^2 + 80.708x + 708.16$	0.84
	3	y = 140.79x + 201.01	0.98	$y = -2.95x^2 + 80.708x + 708.16$	0.84
	4	y = 160.29x + 170.08	0.86	$y = -4.1798x^2 + 121.72x + 283.34$	0.96
	5	y = 85.513x + 713.27	0.84	$y = -1.0933x^2 + 46.441x + 597.71$	0.90

S3: The Fraction of Transpirable Soil Water breakpoint (FTSW Breakpoint), Transpiration Ratio breakpoint (TR Breakpoint) and the initial slope (Slope 1) for the breakpoint regressions between Transpiration Ratio and FTSW for each replicate of each genotype in Experiments 1, 2 and 3. Note, the slope of the line after the breakpoint was forced to be zero.

		Exp. 1			Exp. 2			Exp. 3		
Genotype	Rep	FTSW Breakpoint	TR Breakpoint	Slope 1	FTSW Breakpoint	TR Breakpoint	Slope 1	FTSW Breakpoint	TR Breakpoint	Slope 1
CS 50	1	0.136	1.02	7.12	0.313	1.23	3.23	0.572	1.1	1.69
	2	0.142	0.98	6.16	0.258	1.18	4.19			
	3	0.174	1.06	5.23	0.222	1.02	4.27	0.510	1.2	2.11
	4	0.246	1.10	3.54	0.397	1.05	2.29	0.603	1.2	1.81
	5	0.251	1.19	3.79	0.279	1.18	3.90	0.520	1.3	2.36
CSX2027	1	0.252	0.96	3.11	0.187	0.98	4.80	0.505	1.2	2.30
	2	0.211	0.95	3.80	0.248	0.93	3.27	0.518	1.2	2.13
	3	0.198	0.94	4.01	0.287	0.91	2.73	0.424	1.1	2.35
	4	0.220	0.98	3.67	0.321	0.92	2.37	0.335	0.9	2.40
	5	0.250	0.92	2.84	0.271	0.88	2.65	0.346	0.9	2.08
CSX8521	1	0.139	1.07	6.55	0.257	0.92	2.92	0.449	1.1	2.24
	2	0.151	1.03	6.07	0.262	0.91	2.81	0.531	1.1	1.87
	3	0.162	0.94	4.74	0.139	0.83	4.96	0.293	0.8	2.42
	4	0.162	0.88	4.02	0.223	0.84	3.02	0.333	0.7	1.60
	5	0.191	0.96	3.81	0.266	0.86	2.56	0.268	0.7	1.81
DeltaPEARL	1	0.115	0.99	6.98	0.215	0.85	3.62	0.419	1.0	2.32
	2	0.150	0.97	5.46	0.213	0.85	3.52	0.554	1.1	1.72
	3	0.142	0.96	5.84				0.367	1.1	2.74
	4	0.133	0.90	5.83	0.236	0.88	2.43	0.388	1.1	2.62
	5	0.167	0.89	4.03	0.216	1.12	4.29			
RC-89	1	0.124	0.98	7.19	0.100	0.86	6.91	0.426	1.2	2.57
	2	0.137	0.95	6.08	0.139	0.91	5.45	0.434	1.1	2.22
	3	0.157	0.93	5.27	0.145	0.89	4.89	0.470	1.0	1.80

	4	0.148	0.94	5.20	0.115	0.82	5.48	0.420	1.0	2.10
	5	0.202	0.93	3.20	0.132	0.83	4.30	0.304	0.7	1.76
Sicot 746B3F	1	0.161	0.87	5.28	0.107	0.81	6.62	0.456	1.0	1.94
	2	0.182	0.90	4.52	0.156	0.88	4.72	0.460	1.0	1.83
	3	0.208	0.92	3.61	0.123	0.87	5.03	0.313	0.8	2.13
	4	0.225	0.92	3.13	0.220	0.94	2.90	0.322	0.7	1.63
	5	0.171	0.94	4.33				0.231	0.8	2.37

S4: Regression statistics and equations for each genotype's fitted photosynthesis and conductance responses with respect to the fraction of transpirable soil water (FTSW) content.

	Photosynthesis		
	**r2**	**p- value**	**Equation**
**CS 50**	0.76	<0.0001	y= 40.0/(1+exp(-(x-0.468/0.300))
**CSX2027**	0.5	<0.0001	y= 36.9/(1+exp(-(x-0.260/0.225))
**CSX8521**	0.61	0.0002	y= 54.5/(1+exp(-(x-0.711/0.521))
**DeltaPEARL**	0.38	0.0001	y= 29.4/(1+exp(-(x-0.138/0.185))
**RC-89**	0.31	0.001	y= 28.2/(1+exp(-(x-0.062/0.104))
**Sicot 746B3F**	0.49	<0.0001	y= 37.7/(1+exp(-(x-0.292/0.310))
	**Conductance**		
	**r2**	**p- value**	**Equation**
**CS 50**	0.90	<0.0001	y= 1.23/(1+exp(-(x-0.507/0.116))
**CSX2027**	0.66	<0.0001	y= 1.54/(1+exp(-(x-0.501/0.200))
**CSX8521**	0.6	<0.0001	y= 2.24/(1+exp(-(x-0.833/0.297))
**DeltaPEARL**	0.54	<0.0001	y= 513.8/(1+exp(-(x-3.68/0.461))
**RC-89**	0.35	0.0003	y= 1.06/(1+exp(-(x-0.255/0.216))
**Sicot 746B3F**	0.51	<0.0001	y= 1.38/(1+exp(-(x-0.442/0.223))
